# Supplementary material for: Influence of foetal inflammation on the development of meconium aspiration syndrome in term neonates with meconium-stained amniotic fluid
Source: PeerJ. 2019 May 31;7:e7049. doi: 10.7717/peerj.7049 (PMC6546081; doi:10.7717/peerj.7049)
Supplement: Supplemental Information 2 [file peerj-07-7049-s002.docx]

**Supplemental Table 1: Logistic regression model to explain MAS development using chorioamnionitis, 1-minute Apgar ≤7, and male sex.**

|  | Odds ratio | | | |
| --- | --- | --- | --- | --- |
|  | Mean | 95% Cl | | p-value |
|  |  | Lower | Upper |  |
| Chorioamnionitis | 1.690 | 0.699 | 4.080 | 0.244 |
| 1-minute Apgar ≤7 | 2.220 | 0.907 | 5.320 | 0.081 |
| Male sex | 2.870 | 1.100 | 7.530 | 0.032 |

Chorioamnionitis was not associated with an increased incidence of MAS when adjusted for the Apgar score and sex.

Abbreviations: MAS, meconium aspiration syndrome. CI, confidence interval.
