# Supplementary material for: Influence of foetal inflammation on the development of meconium aspiration syndrome in term neonates with meconium-stained amniotic fluid
Source: PeerJ. 2019 May 31;7:e7049. doi: 10.7717/peerj.7049 (PMC6546081; doi:10.7717/peerj.7049)
Supplement: Supplemental Information 3 [file peerj-07-7049-s003.docx]

**Supplemental Table 2: Multiple linear regression model to explain the total duration of invasive and non-invasive positive airway pressure support using funisitis, 1-minute Apgar ≤7, and male sex.**

|  | Regression coefficient | | | |
| --- | --- | --- | --- | --- |
|  | Mean | 95% Cl | | p-value |
|  |  | Lower | Upper |  |
| Funisitis | 0.567 | -0.294 | 1.428 | 0.194 |
| 1-minute Apgar ≤7 | 0.697 | -0.129 | 1.522 | 0.097 |
| Male sex | 0.753 | -0.080 | 1.586 | 0.076 |

Funisitis was not associated with a longer duration of positive airway pressure support when adjusted for the Apgar score and sex.

Abbreviation: CI, confidence interval.
