# Supplementary material for: Influence of foetal inflammation on the development of meconium aspiration syndrome in term neonates with meconium-stained amniotic fluid
Source: PeerJ. 2019 May 31;7:e7049. doi: 10.7717/peerj.7049 (PMC6546081; doi:10.7717/peerj.7049)
Supplement: Supplemental Information 4 [file peerj-07-7049-s004.docx]

**Supplemental Table 3: Multiple linear regression model to explain the total duration of invasive and non-invasive positive airway pressure support and oxygen supplementation using funisitis, 1-minute Apgar ≤7, and male sex.**

|  | Regression coefficient | | | |
| --- | --- | --- | --- | --- |
|  | Mean | 95% Cl | | p-value |
|  |  | Lower | Upper |  |
| Funisitis | 1.013 | 0.142 | 1.885 | 0.023 |
| 1-minute Apgar ≤7 | 0.329 | -0.229 | 1.442 | 0.439 |
| Male sex | 0.606 | -0.514 | 1.172 | 0.152 |

Funisitis was associated with a longer duration of positive airway pressure support and oxygen supplementation when adjusted for the Apgar score and sex.

Abbreviation: CI, confidence interval.
